# Effects of *Piromyces* sp. CN6 CGMCC 14449 on fermentation quality, nutrient composition and the in vitro degradation rate of whole crop maize silage

**DOI:** 10.1186/s13568-019-0846-x

**Published:** 2019-07-29

**Authors:** Dangdang Wang, Congcong Zhao, Shimin Liu, Tao Zhang, Junhu Yao, Yangchun Cao

**Affiliations:** 1 College of Animal Science and Technology, Northwest A&F University, Yangling, 712100 Shaanxi China; 2 UWA School of Agriculture and Environment, The University of Western Australia, Crawley, WA Australia

**Keywords:** Crop straw, Maize silage, Anaerobic fungi, *Piromyces* Spp., Fibre degradation, Enzymes

## Abstract

This study investigated the effects of the rumen fungus *Piromyces* sp. CN6 CGMCC 14449 as a silage additive on the fermentation quality, nutrient composition and in vitro digestibility of whole crop maize silage. Whole crop maize served as the silage material and was vacuum packed in polyethylene bags. Three ensiling treatments were applied: a control (CK), addition of a fungus (FU) at 10^5^ thallus-forming units per gram, and addition of compound enzyme (EN) at 0.033 mg/g (containing cellulase and xylanase at activities of 90 filter paper units and 6000 IU per gram, respectively). Compared with the CK, the FU and EN treatments decreased the pH after 30 days fermentation (*P *<0.05). Both FU and EN treatments increased the lactate, crude protein, and water-soluble carbohydrate contents (*P *<0.05), whereas reduced the acetate, ADF and NDF contents as well as the ammonia nitrogen to total nitrogen ratio in silage after 30 days of ensilaging (*P *<0.05), compared with those for the CK, while no changes were found in the dry matter and dry matter recovery (*P* > 0.05). The fungal inoculant increased the in vitro digestibility of dry matter, NDF and ADF in silage after 30 days fermentation (*P *<0.05). In conclusion, the rumen fungus *Piromyces* sp. CN6 CGMCC 14449 can improve the quality and nutrient composition of whole crop maize silage and increase the crude fibre digestibility.

## Introduction

Crop straws are very abundant and renewable lignocellulosic biomass resources that can be used as feed materials for ruminant animals. 840 million tons crop straws per annum are produced in China, and only about 22–28% of the straws is used as feedstuff (Jiang et al. 2012; Long et al. 2013). This is a great waste considering that China has limited supply of feedstuffs to support its livestock industries. Factors that high fibre content, poor palatability, and low digestibility of crop straws limit its utilization in the feedstuff industry. Adding a cellulase-secreting fungus or cellulase during the ensiling process can degrade plant cell walls, improve the nutritional quality and reduce crude fibre content of silages, thereby increasing the rate of forage utilization by ruminant animals (Refat et al. 2018; Robledo et al. 2016).

Anaerobic fungi (Neocallimastigomycota), the only obligate anaerobic microorganisms in the fungal community, play an important role in the degradation of roughage (Bootten et al. 2011; Henske et al. 2018). Anaerobic fungi not only degrade hard-to-degrade fibrous tissues through the growth of rhizoids, but also secrete a series of highly active plant cell wall degradation enzymes for synergistically degrading complexly structured plant cell walls (Brunecky et al. 2013; Haitjema et al. 2014). Studies have shown that the activity of the enzymes produced by rumen fungi and capacity of degrading the plant cell wall are higher than these of the enzymes produced by *Trichoderma reesei*, a widely used strain, and by *Aspergillus nidulans*, which is currently used in industry for cellulase production; in particular, the xylanase activity of the *Piromyces* fungal genus is more than three times that of *Trichoderma* and *Aspergillus* (Solomon et al. 2016). Thareja et al. (2006) showed that rumen anaerobic fungi could increase the in vitro degradation rates of dry matter (DM) and neutral detergent fibre (NDF) of wheat straw. Adding pure cultured rumen fungi during ensiling rice straw can not only reduce the content of NDF and acid detergent fibre (ADF), but also increase the crude fibre degradation rate (Lee et al. 2015). In addition, under conditions imposed by a highly coarse feed, direct gavage of rumen fungi can increase the daily weight gain of fattening cattle and the milk production of lactating cattle, while simultaneously increasing the concentration of volatile fatty acids (VFAs), zoospore numbers and feedstuff utilization in the rumen (Lee et al. 2000; Saxena et al. 2010; Tripathi et al. 2007). As a group of fungi functionally important in plant cell wall degradation, anaerobic fungi can efficiently degrade agricultural biomass resources, such as maize and wheat straws. This capability has gained anaerobic fungi and their plant cell wall degradation enzymes increasingly wide application prospects for alleviating feedstuff shortages in animal husbandry and for improving animal roughage utilization.

We therefore, hypothesized that using anaerobic fungi as an ensilaging additive can improve the quality of silage made of crop straws. In this study, the effects of the rumen fungus *Piromyces* sp. CN6 CGMCC 14449 as a silage additive were examined on the fermentation quality, nutrient composition and in vitro degradation rate of whole crop maize silage.

## Materials and methods

### Silage raw materials

Whole crop maize (Zhongyuandan 32) was cultivated at the Animal Husbandry Teaching and Experimental Base of Northwest A&F University in Yangling, Shaanxi, China (34°17′N, 108°04′E, altitude 518 m a.s.l.). This region is characterised by hot, humid subtropical summer, and cold, dry winter. Mean total annual rainfall is 542 mm, most of rains is delivered from July to late October. Whole crop maize was harvested at the milk line stage, with the moisture content of 65.66 ± 1.55%, and then chopped to segments 2–5 cm long for ensilaging. The composition of whole crop maize is shown in Table 1.

**Table 1:** The nutrient composition (g kg^−1^ DM, unless stated otherwise) of whole crop maize

Ingredient	DM (g kg^−1^ FM)	CP	EE	WSC	NDF	ADF
Whole crop maize	343.4 ± 15.5	70.2 ± 1.2	27.5 ± 6.9	82.4 ± 4.5	563.8 ± 13.6	310.3 ± 8.7

FM: Fresh weight; DM: dry matter; CP: crude protein; EE: ether extract; WSC: water soluble carbohydrate; NDF: neutral detergent fibre; ADF: acid detergent fibre

### Complex enzyme preparation

A silage-specific additive produced by Snow Brand Seed Ltd (Sapporo, Japan) was used. The additive was in a solid form and contained cellulase and xylanase at respective activities of 90 filter paper units per gram (FPU/g) and 6000 international units per gram (IU/g).

### Fungal inoculants

The strain *Piromyces* sp. CN6 CGMCC 14449 (GenBank: KY368105.1) was an anaerobic fungus isolated from the rumen of Xinong Saanen dairy goats, and this strain was characterized with a monocentric nature of growth, uniflagellated zoospores, and extensive and filamentous rhizomycelia, and had the highest enzyme activity for the degradation of plant cell walls among a total of 12 fungal strains (Wang et al. 2017). The fungal preparation for experimentation was the culture for 5 days after with fungus at > 10^6^ thallus-forming units per millilitre (TFUs mL^−1^). The fibrolytic enzymes activities, pH optima and pH stability of the preparation were determined by the 3,5-dinitrosalicylic acid method (Wei et al. 2016; Yue et al. 2009). Briefly, the xylanase, CMCase and microcrystalline cellulose activity were determined using the dinitrosalicylic acid method with the specific substrates of xylan, carboxymethyl cellulosedodium and microcrystalline cellulose respectively. Acetyl esterase activity was assayed by measuring the amount of *p*-nitrophenol released from 2 mmol/L *p*-nitrophenyl acetate (Alfa Aesar Chemicals) in PBS. The assays were performed in 96-well reaction microplates and a multimode reader (Synergy HT, BioTek Instruments, Winooski, USA) according the manufacturer’s guidelines. The enzyme activities per millilitre of fungal prepation broth were as follows: 1655 milli-enzyme units (mU) for xylanase activity, 93 mU for carboxymethyl cellulase activity, 51 mU for microcrystalline cellulase activity and 153 mU for acetylesterase activity. Xylanase displayed an optimum pH of 5.0 and remained more than 80% activity at pH 4.0 and pH 7.0. Xylanase and acetylesterase were stable within the pH range from 4.5 to 8.0.

### Experimental design

There were three ensilaging treatments in this experiment, a control (CK), addition of fungus (FU) preparation and addition of complex enzyme (EN) product for ensilaging whole maize crop. For the CK, 100 mL distilled water was uniformly sprayed on the silage material; for the FU treatment, 100 mL of the anaerobic fungi preparation was uniformly sprayed, so each kg of ensiled crop maize contained 165.5 IU of xylanase activity, 9.3 IU of carboxymethyl cellulase activity, 5.1 IU of microcrystalline cellulase activity and 15.3 IU of acetyl esterase activity; for the EN treatment, 100 mL of distilled water containing 33 mg of the compound enzyme product as uniformly sprayed, and each kg maize crop contained 198 IU of xylanase activity and 2.3 FPUs of cellulase activity. To examine the course of changes in silage quality, the ensiling fermentation was stopped after 10, 30 and 60 days for sampling. Therefore, the experiment formed a 3 × 3 factorial design, i.e., 3 treatments crossed with 3 ensiling durations. There were five replicates for each treatment in each duration, so a total of 45 replicates (3 × 3 × 5) were completed. We determined to open the bags at day 10, 30 and 60 for these reasons: the count of added anaerobic fungi continued declining with the pH value of silage, remained greater than that without adding fungi when the pH was above 4.7, but almost diminished with the pH value below 4.5 (Lee et al. 2015); and the pH value of whole crop maize silage with initial 35% dry matter (similar to the maize crop used in this experiment) can drop to 4.4 after ensiling for 8 days (Filya 2004). Therefore, 10 days was determined to examine fungus activity in the silage in screening electron microscopy. After 15 days fermentation, the pH value of whole crop maize silage reached 4.0 or 3.75 after 49 days and there were a little or no change afterwards (Filya 2004; Neureiter et al. 2005), indicating that the fermentation almost stops at the low pH, so we opened the bags on days 30 and 60 for examine the nutritive value of the silage.

For each replicate, 1 kg of chopped whole maize crop was ensiled in a polyethylene bag. The crop was quickly packaged inside the bag, then the bag was vacuum-sealed with a vacuum packaging machine and stored in an anaerobic workstation at ambient temperature (about 24 °C). After fermentation for 10, 30 and 60 days, 5 bags per treatment were unsealed, and the surface silage was discarded. The rest of silage was thoroughly mixed, and samples were taken and immediately stored at − 80 °C for further assessment.

### Measurements and analysis

#### Scanning electron microscope observation

The stems and leaves of whole crop maize ensilaged for 10 days were treated according to the method in Rezaeian et al. (2004). The surface damage and fungal attachment to the leaves were then observed by scanning electron microscopy (Hitachi, S-4800, Hitachi, Tokyo, Japan).

#### Silage quality

Ten grams of silage sample was weighed and placed into a 100-mL Erlenmeyer flask, into which 90 mL of distilled water was added, and thoroughly mixed. The flask was then sealed with film and let stand at 4 °C for 24 h. The sample was filtered with four layers of gauze and qualitative filter paper to obtain liquid extracts (Han et al. 2004), which was used for the determination of pH, ammonia nitrogen (NH_3_-N) and organic acids. The pH was measured with a glass electrode pH meter (Mettler Toledo, Zurich, Switzerland). The contents of lactic, acetic, propionic and butyric acids were measured in high-performance liquid chromatography (Hitachi L-2000, Hitachi, Tokyo, Japan) according to the method described by Ohmomo et al. (1993). The NH_3_-N content was determined with the phenol-hypochlorite colorimetric method (Broderick and Kang 1980).

#### Nutrient composition of silage

The silage was sampled according to Young et al. (2012). DM, crude protein (CP), water-soluble carbohydrate (WSC) and ether extract (EE) contents as well as the NDF and ADF contents were determined by conventional methods (AOAC 2005; Van Soest et al. 1991). Briefly, DM content was determined by oven drying after 72 h at 50 °C, while the total N content was determined using an elemental analyser (LECO TruSpec N, St. Joseph, MI). NDF and ADF were sequentially determined using an ANKOM Fiber Analyzer (ANKOM Technology, Macdon, USA). The method of Wardynski et al. (1993) was used to calculate the dry matter recovery rate (DMR) as the ratio of the total DM amount after ensiling to that before ensiling.

#### In vitro fermentation of silage

The in vitro digestibility analysis for the silage DM, NDF and ADF were measured at 30 days after fermentation with an in vitro incubator by referring to and slightly modifying the method described by Adesogan (2005). F57 ANKOM filter bags (ANKOM Technology, Macedon, NY, USA) were prewashed with acetone for 3–5 min and then dried completely. Then approximately 0.5 g of shredded and air-dried silage sample was placed in a filter bag and weighed. The bag was sealed and placed in a digestion vessel (Daisy II incubator) preheated to 39 °C. Each vessel received 23 sample-containing filter bags and two blank filter bags, which were evenly distributed on both sides of a divider plate in each vessel. The following were then quickly added to the digestion vessel: 1600 mL of ruminal buffer preheated to 39 °C and 400 mL of rumen fluid that had been filtered through four layers of gauze. The digestion vessel was immediately placed in the incubator, and the heating and rotation switches were turned on. After 48 h of incubation, the digestion vessel was drained, and each filter bag was rinsed with cold water until the bag was clean. The rinsed filter bag was then placed in a fibre analyser to measure the NDF and ADF contents of the sample. Finally, the in vitro degradation rates of DM (IVDMD), NDF (IVNDFD), and ADF (IADFD) in the silage were receptively calculated for using the following equation:

$$ {\text{The}}\;{\text{in}}\;{\text{vitro}}\;{\text{degradation}}\;{\text{rate}}\;(\% ) = 100 - \left( {{\text{m}}2 - {\text{m}}1 \times {\text{C}}1} \right)/{\text{m}} \times 100, $$

where m = weight (g) of DM, NDF or ADF in sample, m1 = filter bag weight (g), m2 = weight (g) of filter bag plus the residue of DM, NAD, or ADF, and C1 = ratio of blank bag weight after to before in vitro digestion.

### Statistical analysis

The data measured for one ensiling duration (i.e., on 30 days fermentation) were analysed by a one-way ANOVA procedure (SPSS 20.0) to test the differences among the three treatments, and these measures included the nutrient concentrations, and the degradability of DM, NDF and ADF of the silage. For these dates measured in three ensiling durations, such as the pH values, and NDF and ADF contents of the silage, a two-way factorial ANOVA was applied to examine the effects of treatment, ensiling duration and their interaction. The data was shown as mean ± SD. Significance was declared at *P *<0.05.

## Results

### Scanning electron microscopic observation

**Fig. 1:**
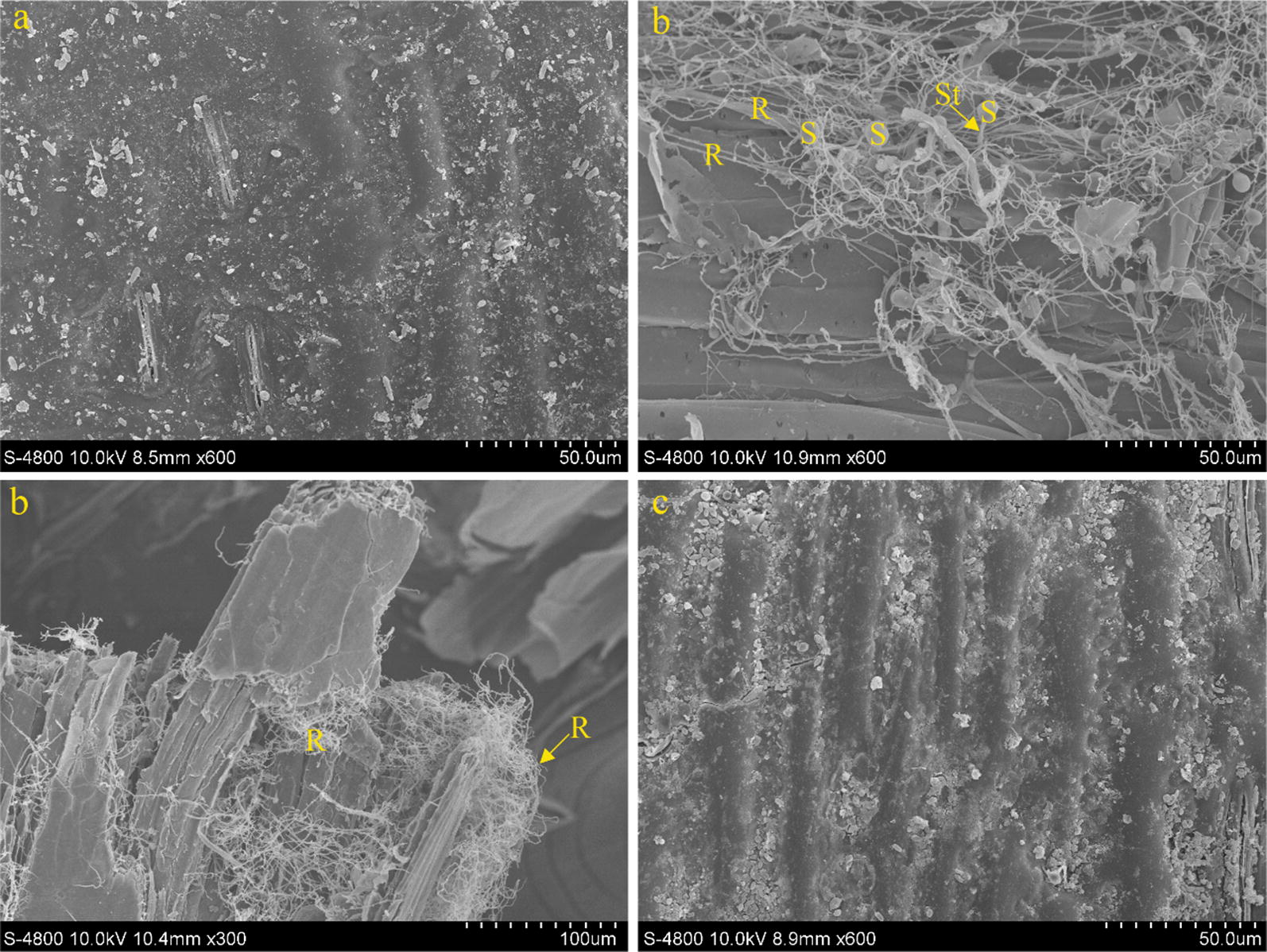
Images of scanning electron micrograph of whole crop maize silage on 10 days ensilaging. S, fungus sporangium; St, fungus sporangiophore; R, fungus rhizoid. **a** Control; **b** fungus addition; **c** compound enzyme addition

Figure 1b illustrates that a large number of rumen anaerobic fungi were attached to the stem and leaf surfaces of the silage on 10 days of fermentation. Meanwhile, compared to the CK (Fig. 1a) and EN (Fig. 1c), the whole crop maize in the FU (Fig. 1b) had relatively rough surfaces. Rumen anaerobic fungi were observed to multiply during the early period of ensilaging and to successfully attach to the stem and leaf surfaces.

### Effect of different treatments on the fermentation quality of silage

Table 2 shows the pH value, ADF and NDF contents of the silage up to 60 days fermentation. On day 10 and 30 fermentation, the pH of the EN treatment was lower than that of the CK (*P *<0.05), and the pH of the FU treatment appeared slightly lower than that of the CK, but the difference was not significant (*P *>0.05). At 60 days of fermentation, no significant difference in silage pH existed among the three treatments (*P *>0.05). There was significant interaction between the treatment and ensiling duration, and the decline of pH with the duration was faster from day 10 to day 30 in the FU treatment, compared with the other two treatments. Both FU and EN treatments showed significantly lower contents of NDF and ADF in silage fermented for 10, 30 and 60 days, compared with the CK (*P *<0.05). However, there were no differences in the pH, NDF and ADF contents between FU and EN treatments on both day 30 and 60 (*P* > 0.05), indicating there were no further changes in NDF and ADF degradation from day 30 to day 60 of fermentation. For this reason, we did not examine the nutritive values of the silage fermented for 60 days, because this research focused mainly on the effects of the fungus and enzyme additives on fibre degradation of silage.

**Table 2:** Effects of different additive treatments and ensiling durations on the pH value, NDF and ADF contents (g kg^−1^ DM) of whole crop maize silage

Ensiling duration	Treatment	pH	NDF	ADF
10 days	CK	4.31^a^	554.1^a^	318.8^a^
	FU	4.24^a^	539.0^b^	301.8^b^
	EN	4.08^b^	540.1^b^	298.2^b^
30 days	CK	3.79^a^	546.6^a^	301.1^a^
	FU	3.64^b^	509.7^b^	283.4^b^
	EN	3.62^b^	517.7^b^	289.0^b^
60 days	CK	3.72	543.3^a^	309.2^a^
	FU	3.65	498.3^b^	288.7^b^
	EN	3.68	516.8^b^	282.6^b^
SEM		0.04	3.3	2.3
Main effect means				
Ensiling duration	10 days	4.21^a^	544.4^a^	306.3^a^
	30 days	3.69^b^	524.9^b^	291.2^b^
	60 days	3.68^b^	519.5^b^	293.5^b^
Treatment	CK	3.94	548.0^a^	309.8^a^
	FU	3.84	515.7^b^	291.3^b^
	EN	3.79	524.9^b^	290.0^b^
Source of variation *P* value				
	Ensiling duration	<0.001	<0.001	0.001
	Treatment	<0.001	<0.001	<0.001
	D*T	0.034	0.196	0.646

CK: control; FU: fungus addition; EN: compound enzyme addition. NDF: neutral detergent fibre; ADF: acid detergent fibre; T: effect of treatment; D: effect of ensiling duration; SEM: standard error of the means

Mean values with different superscript letters in the same column differ significantly (*P *< 0.05). The interaction between treatment and ensiling duration is significant (*P *< 0.05), so main effect means are not present. Because of no significant interactions on NDF and ADF contents (*P *> 0.05), the main effect means are presented, and the differences among the means were examined

### Effects of different treatments on nutrient substances in silage

Table 3 indicates that at 30 days of fermentation, both FU and EN treatments had higher content lactate, lower acetate, so higher ratio of lactate to acetate, and lower the NH_3_-N/total nitrogen ratio in the silage (*P *<0.05) compared with those in the CK. No butyric acid was detected in any of the three treatments.

**Table 3:** Effects of different additive treatments on the fermentation quality (g kg^−1^ DM, unless stated otherwise) of whole crop maize silage after 30 days fermentation

	Treatment*			SEM	*P*-value
	CK	FU	EN		
Lactic acid	57.6^b^	75.0^a^	80.5^a^	3.7	0.016
Acetic acid	16.7^a^	12.9^b^	14.0^b^	0.7	0.033
Butyric acid	ND	ND	ND	–	–
Lactic acid/acetic acid (%)	3.41^b^	5.94^a^	5.90^a^	0.41	0.005
NH_3_-N/total nitrogen (%)	7.35^a^	5.96^b^	5.88^b^	2.51	0.012

CK: control; FU: fungus addition; EN: compound enzyme addition; SEM: standard error of means; ND: not detected

*Mean values with different superscript letter within the row differ significantly (*P *< 0.05)

As shown in Table 4, both FU and EN treatments showed significantly higher protein and water soluble carbohydrate contents (*P *<0.05), and lower NDF and ADF contents (*P *<0.05) in silage fermented for 30 days, compared with those in the CK. No significant difference existed among the three treatments for the silage DM, EE content and dry matter recovery rate in the silage fermented for 30 days (*P *>0.05).

**Table 4:** Effects of different additive treatments on the nutrient value (g kg^−1^ DM, unless stated otherwise) of whole crop maize silage after 30 days fermentation

	Treatment*			SEM	*P*-value
	CK	FU	EN		
DM (g kg^−1^ FM)	263.6	264.8	255.2	2.9	0.355
NDF	546.6^a^	509.7^b^	517.7^b^	5.4	0.017
ADF	301.1^a^	283.4^b^	289.0^b^	3.0	0.035
CP	80.8^b^	87.0^a^	86.2^a^	1.1	0.011
EE	32.5	29.4	28.9	0.9	0.192
WSC	37.5^b^	49.3^a^	50.5^a^	2.5	0.034
DMR	959.6	964.5	941.5	6.1	0.302

DM: dry matter; FM: fresh matter; NDF: neutral detergent fibre; ADF: acid detergent fibre; CP: crude protein; EE: ether extract; WSC: water soluble carbohydrate; DMR: dry matter recovery rate; SEM: standard error of means; CK: control; FU: fungus addition; EN: compound enzyme addition

*Mean values with different superscript letter within the row differ significantly (*P *< 0.05)

### Effects of different treatments on the in vitro degradation rate of silage

Table 5 indicates that with fermentation for 30 days, the FU treatment significantly increased silage IVDMD and IVADFD (*P *<0.05), and both FU and EN treatments increased IVNDFD (*P *<0.05), compared with those in the CK.

**Table 5:** Effects of different additive treatments on the in vitro degradation rate (g kg^−1^ DM) of DM, NDF, and ADF in whole crop maize silage after 30 days fermentation

	Treatment*			SEM	*P*-value
	CK	FU	EN		
IVDMD	612.3^b^	651.2^a^	628.8^ab^	5.4	0.011
IVNDFD	481.8^b^	525.3^a^	526.8^a^	8.3	0.026
IVADFD	427.1^b^	477.5^a^	440.2^ab^	8.7	0.040

IVDMD: In vitro dry matter digestibility; IVNDFD: in vitro neutral detergent fibre digestibility; IVADFD: in vitro acid detergent fibre digestibility; SEM: standard error of means; CK: control; FU: fungus addition; EN: compound enzyme addition

*Mean values with different superscript letter within the row differ significantly (*P *< 0.05)

## Discussion

Silage fermentation achieves the purpose of feedstuff preservation in that under anaerobic conditions, and lactic acid is produced from fermentation of soluble carbohydrates by lactic acid bacteria, which can eventually reduce the silage pH below 4.2 and thereby inhibit the activities of various microorganisms (Kleinschmit et al. 2005). In the present experiment, the pH of whole crop maize silage fermented for 30 days dropped below 3.8 in all three treatments, and the further but not significant drops occurred at day 60 fermentation. Kristensen et al. (2010) studied the effects of homofermentative inoculation (*Lactobacillus pentosus* and *Pediococcus pentosaceus*) and heterofermentative inoculation (*Lactobacillus buchneri*) on whole crop maize silage and reported a pH below 4.0 in all the silage treatments. The vast majority of silage successfully prepared from whole crop maize showed a pH below 4.0 (Bedrosian et al. 2012; Li and Nishino 2011; Schmidt and Kung. 2010). This pattern indicates relatively successful silage production in the present experiment.

In this present study, the maize silage added with both the fungus inoculants and complex enzyme had higher concentrations of lactic acid and lower concentrations of acetic acid and NH_3_-N than the control, which can be significant for the stability of silage. The results are in agreement with the relatively lower pH in these two treatments in this study. The results show that the maize silage treated with the fungus and enzymes produce a more homolactic fermentation. The findings are in agreement with those of Driehuis et al. (2001), Kleinschmit and Kung (2006).

Rumen anaerobic fungi can not only grow rhizoids that penetrate the stratum corneum and lignin of plant cell walls but also produce highly active cellulases, hemicellulases, esterases and cellulosomes that degrade plant cell walls (Brunecky et al. 2013; Dijkerman et al. 1996; Wang et al. 2011). The present study found that adding the fungus inoculants and complex enzyme for ensilage of whole crop maize did not alter the DM content and the DMR, but reserved more protein and WSC while consumed more NDF and ADF in the silage. This outcome shows the rumen anaerobic fungi and compound enzymes did lead to the conversion of plant polysaccharides to more WSCs, in agreement with Nagpal et al. (2009), Ranganathan et al. (2017) and Li et al. (2018) who reported the anaerobic fungus and its fibrolytic enzymes could decompose lignocellulosic biomass into WSCs.

Proteolysis in silage has been mainly considered a result of the plant and microorganisms proteolytic enzymes, and NH_3_-N concentration in silage is the main indicator of protein degradation (Guzatti et al. 2017). In this study, both FU and EN treatments had lower the NH_3_-N/total nitrogen ratio in the silage compared with that in CK treatment, which indicated that the degradation of true proteins into non-protein nitrogen was low (Hoffman et al. 2011). Thus, CP in FU and EN treatments was significantly higher than that in CK treatment.

In addition, this experiment demonstrated that rumen fungi improve the IVDMD, IVNDFD and IVADFD of silage. These results are consistent with data from Paul et al. (2010) and Nagpal et al. (2011) who reported that anaerobic fungi increased the crude fiber degradability on in vitro fermentation test. The result may have occurred because anaerobic fungi and their degrading enzymes can destroy the texture of straws and reduce NDF and ADF contents of silage, facilitating straw degradation by rumen microorganisms and enzymes during in vitro digestion. Comino et al. (2014), Weinberg et al. (2007) and Opsi et al. (2013) reported some lactic acid bacterial inoculants, esterase-producing inoculants or fibrolytic enzymes could breakdown hemicellulose structure during ensiling, which may improve NDF or ADF digestibility, but no change or even decrease of the residual NDF. This illustrates that anaerobic fungi as a silage inoculation have more promising effects on improving crude fibre degradation.

Furthermore, the observation by scanning electron microscopy of the surface of silage fermented for 10 days after its inoculation with rumen anaerobic fungi detected successful fungal attachment to the stem and leaf surfaces of the straw. Lee et al. (2015) also found that anaerobic fungi could survive when the pH of rice straw fermentation exceeded 4.7 and that a large number of anaerobic fungi were observable on the rice straw surfaces. This result showed that anaerobic fungi proliferated greatly during the early stage of silage fermentation and destroyed the plant cell wall surface through the growth of rhizoids. Previous studies showed that the plant-wall-degrading enzymes secreted by anaerobic fungi retained their degradative function under acidic conditions (Morrison et al. 2016; Ranganathan et al. 2017), which provides a theoretical basis for the degradation of plant cell walls by anaerobic fungi under ensiling conditions.

Presently, compound enzyme preparations, such as cellulase and xylanase, have been applied in the field of silage fermentation and have achieved good results. Dean et al. (2005) and Xing et al. (2009) found that the addition of fibrolytic enzymes during the ensiling process can reduce the pH, ADF and NDF contents, thereby improving the silage fermentation quality. In this present study, after whole crop maize was ensiled for 30 days, the silage NDF and ADF contents were decreased for the FU treatment respectively by 9.59% and 8.67%, while decreased by 8.98% and 6.86% for the EN treatment. The present experiment showed that the anaerobic fungi exerted the effects equivalent to those of the compound enzyme preparation on degradation of NDF and ADF in ensiling process of whole crop maize.

Conclusively, these results illustrated that the addition of the rumen fungus *Piromyces* sp. CN6 CGMCC 14449 for ensilaging whole crop maize can improve silage quality and nutrient composition, and increase the silage crude fibre degradation rate.

## Data Availability

The dataset supporting the conclusions of this article is included within the article. All data are fully available without restriction.

## Abbreviations

DM: dry matter
NDF: neutral detergent fibre
ADF: acid detergent fibre
VFAs: volatile fatty acids
CP: crude protein
WSC: water-soluble carbohydrate
EE: ether extract
IVDMD: in vitro dry matter digestibility
IVNDFD: in vitro neutral detergent fibre digestibility
IVADFD: in vitro acid detergent fibre digestibility
